# A Case of Lateral Lymph Node Dissection for Recurrence of Rectal Cancer with Senhance Surgical System

**DOI:** 10.70352/scrj.cr.25-0009

**Published:** 2025-06-24

**Authors:** Yume Minagawa, Yasuhiro Ishiyama, Sohei Akuta, Misuzu Yamato, Akihito Nakanishi, Hisashi Hayashi, Takatsugu Fujii, Naoto Okazaki, Chikashi Hiranuma, Yasumitsu Hirano

**Affiliations:** Department of Gastroenterological Surgery, Saitama Medical University International Medical Center, Hidaka, Saitama, Japan

**Keywords:** Senhance Surgical System, lateral lymph node dissection, rectal cancer

## Abstract

**INTRODUCTION:**

We have performed approximately 200 colorectal cancer surgeries using Senhance Surgical System in our department and we have standardized the surgical procedure with Senhance. For the first time, we report the case of lateral lymph node dissection for recurrence of rectal cancer with Senhance Surgical System.

**CASE PRESENTATION:**

The patient was a 64-year-old female who was diagnosed with lateral lymph node recurrence of rectal cancer. We performed right lateral lymph node dissection with Senhance Surgical System. The operation time was 175 min and blood loss was 5 mL, with no intraoperative complications. The final histopathologic diagnosis was metastasis of rectal cancer.

**CONCLUSIONS:**

We present the first lateral lymph node dissection performed using the Senhance Surgical System. We will accumulate more cases and study its usefulness in the future.

## Abbreviations

CA19-9
: carbohydrate antigen19-9
CEA
: carcinoembryonic antigen

## INTRODUCTION

The Senhance Surgical System (Asensus Surgical, Durham, NC, USA) was introduced at our hospital in 2017.^1–5)^ The number of colorectal cancer surgery using Senhance Surgical System will exceed 200 colorectal cancer surgeries in 2024 in our department. We have standardized the surgical procedure with Senhance.^6)^ This system was developed as an extension of laparoscopic surgery and it has advantages in terms of protective tissue grasp, tactile feedback system, and eye-tracking camera.

We expect that Senhance is suitable for lymph node dissection in particular, due to its superiority of 3-mm forceps in protective tissue grasping, so we actively perform “Fusion Surgery” such as lymph node dissection with the Senhance and mobilization with laparoscopic surgery.

For the first time, we report the case of lateral lymph node dissection for recurrence of rectal cancer with Senhance Surgical System.

## CASE PRESENTATION

**Fig. 1:**
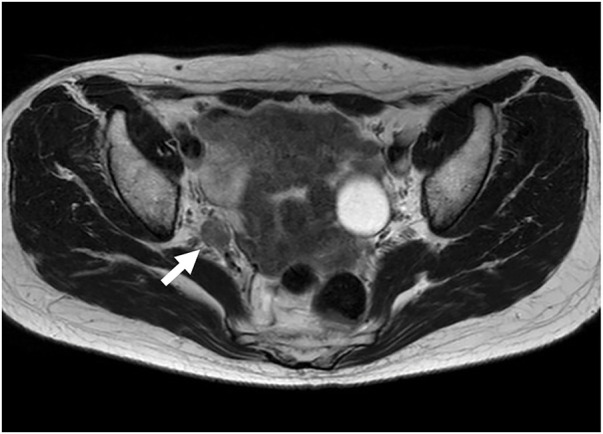
MRI finding. MRI scan showed enlargement of the right lateral lymph node (white arrow), whose region was distal of the internal iliac artery, and whose diffusion-weighted images showed a high signal.

The patient was a 64-year-old female who had a previous laparoscopic low anterior resection with transanal total mesorectal excision (taTME) for lower rectal cancer 4 years ago. The operation time was 217 min and blood loss was minimal, with no intraoperative complications. The pathological diagnosis was Stage I. Follow-up was every 3 months on an outpatient basis. CT and MRI scans showed enlargement of the right lateral lymph node whose region was distal of the internal iliac artery (#263D, **Fig. 1**). The positron emission tomography (PET)-CT scan showed no other intakes except for that of the lymph node. Tumor markers CEA, CA19-9 were 1.6 ng/mL and 11.7 U/mL. Her body mass index was 20.3 kg/m^2^ and she had no past medical history except rectal cancer.

We performed right lateral lymph node dissection with Senhance Surgical System.

### Surgical Procedure

**Fig. 2:**
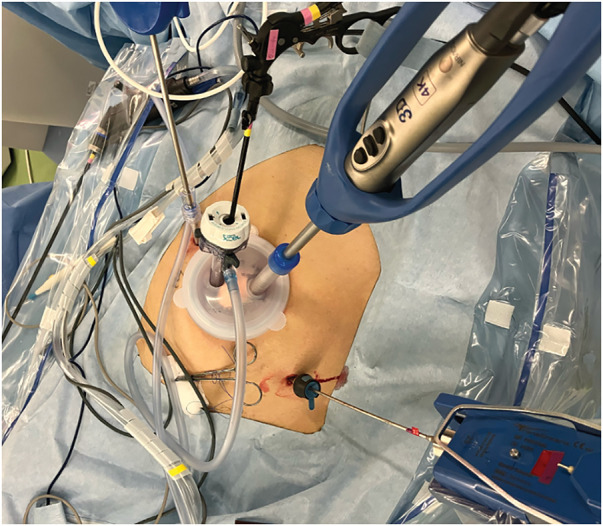
Port Placement. A 3-cm longitudinal incision at the umbilicus. A 12-mm camera port and another 12-mm port were introduced. A 3-mm port was inserted on the left side of the abdomen and a 5-mm port on the right side of the abdomen.

**Fig. 3:**
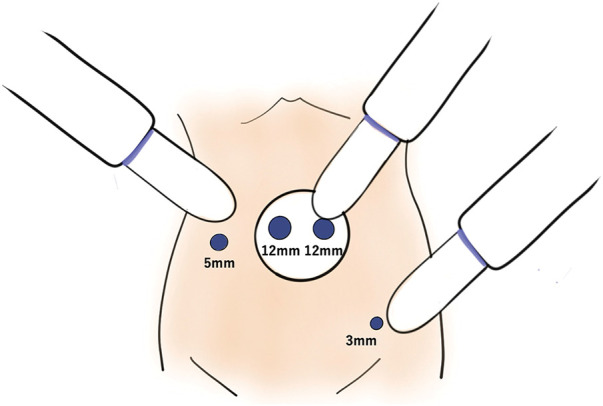
Port placement.

**Fig. 4:**
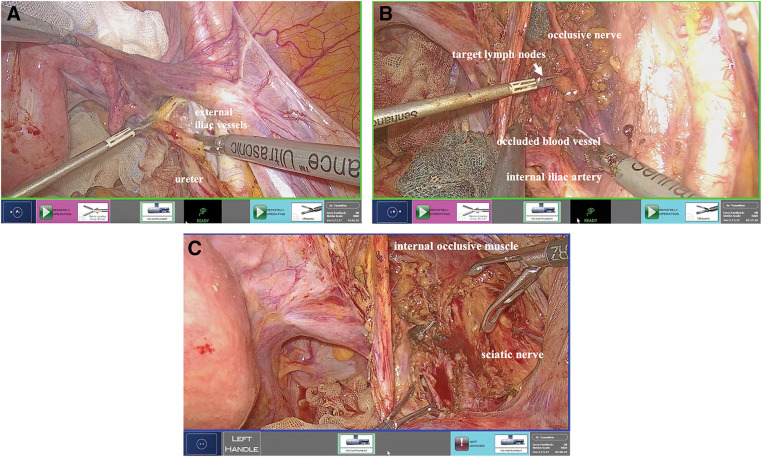
(**A**–**C**) Intraoperative findings. The target enlarged lymph node was dissected.

The patient was placed in lithotomy position under general anesthesia for a mini-laparotomy, performed via 3-cm longitudinal incision at umbilicus. A 12-mm camera port and another 12-mm port were introduced by LAP PROTECTOR (Hakko, Tokyo, Japan). A 3-mm port was inserted on the left side of the abdomen and a 5-mm port with Ultrasonic was inserted on the right side of the abdomen, shown in **Figs. 2** and **3**. The uterus and ovaries were suspended with 2-0 prolene sutures. The peritoneum was incised, the ureter was mobilized laterally, the external iliac artery and vein were exposed, and the lymph nodes on the anterior side were resected. The anterior sides of the psoas major and obturator internus muscles were exposed, the lymph nodes were resected along the obturator nerve, and the obturator artery and vein were clipped and separated. The target enlarged lymph node was found on the medial side of the umbilical artery. The superior vesical artery was clipped and separated, adipose tissue was attached to the resected side along the obturator internus muscle, and the lymph node was dissected (**Fig. 4**).

Hemostasis was confirmed by irrigation and a drain was placed in the lateral cavity from the right lower abdomen.

The operation time was 175 min and blood loss was 5 mL, with no intraoperative complications. The postoperative course was normal. The patient was discharged on the 7th postoperative day. The final histopathologic diagnosis was metastasis of rectal cancer.

## DISCUSSION

Recent advancements in robotic-assisted surgery systems have brought remarkable innovations to the field of surgical medicine. Robotic surgery, now gaining widespread adoption globally, is attracting attention as a technology that offers more precise and safer techniques compared with traditional laparoscopic and open surgeries. Notably, the automatic control of hand tremors and the provision of detailed visual information via 3D cameras enable more accurate and delicate maneuvers, facilitating minimally invasive procedures that benefit patients. Additionally, for the surgeon, the ability to sit at a console and maintain focus during long surgeries while reducing fatigue is a significant advantage. This mitigates the physical burden on the surgeon, a challenge often associated with conventional surgeries.

Our institution was the first in Japan to introduce the Senhance Surgical System in 2017, and we have since performed approximately 200 colorectal cancer surgeries using this system. One of the distinctive features of Senhance, in contrast to other robotic surgery systems, is its haptic feedback function.^7)^ This allows the surgeon to perceive real-time tactile information during the procedure, enabling more precise and safer surgery by sensing tissue stiffness and resistance. This tactile feedback played a critical role, particularly in the delicate operation of lateral lymph node dissection performed in this case.

Senhance has already been adopted in many facilities across Europe and the United States,^8,9)^ with numerous reports confirming its safety and efficacy.^10)^ In Japan as well, the use of Senhance for colorectal cancer surgeries is gradually expanding^6)^ and its range of applications continues to grow. We are pursuing further advancements in minimally invasive surgery, including reduced-port surgery^3–5)^ and lateral lymph node dissection using Senhance. Senhance and laparoscopic surgery for colon cancer, data from our institution show that Senhance makes it possible to perform surgery with comparable short- and mid-term results compared with laparoscopic surgery.^11)^ Although lateral lymph node dissection using the da Vinci has become widely used, in Japan, insurance coverage is limited to cases in which the procedure is performed in conjunction with rectal cancer surgery.

Therefore, when performing lateral lymph node dissection for a recurrent mass of rectal cancer as in this case, the da Vinci is not covered by insurance, and the Senhance is the only robot that is currently covered by insurance for this procedure.

We selected Senhance for this case in order to enjoy its superiority in terms of surgeon ergonomics, stability of the surgical field, and precision forceps manipulation, which are characteristics of robotic surgery, compared with laparoscopic lateral dissection of the right side. In the lateral lymph node dissection performed in this case report, the precise handling capabilities of Senhance were effectively utilized, allowing the lymph node dissection to be completed without damaging fatty tissues or lymph nodes. The use of ultrasonic coagulating shears minimized the need for frequent instrument changes, contributing to a reduction in overall surgery time.

In addition, lymphocele formation is one of the postoperative complications of lateral dissection and is often difficult to treat.^12)^ In Senhance-assisted lateral dissection, the ultrasonic coagulation shears can be used to prevent the formation of lymphocele.

Furthermore, by using a 5-mm port for suction operated by the assistant, the need for additional ports was eliminated, thus minimizing the postoperative burden on the patient. With only three incision sites, the patient experienced minimal postoperative pain, which likely contributed to faster recovery and improved postoperative satisfaction.

However, some challenges with the Senhance system were also observed. Specifically, there are limitations in the range of motion of the robotic arms, which made instrument manipulation difficult in cases involving patients with a narrow pelvis or in cases with obese patients. Additionally, as this was our first attempt, the optimization of port placement and the time required for docking affected the surgical process. Nevertheless, these issues can be resolved with further experience, and maximizing the unique advantages of Senhance will be a key objective moving forward. Limitations in the range of motion of the forceps in Senhance may be improved to some extent by selecting an optimal port insertion position and pre-adjusting the bed height.

Many of the limitations that cannot be improved by these means are often due to the distance from the port where the forceps is inserted to the connection site of the robotic arm of the forceps, and we believe that these limitations can often be addressed by developing forceps with shorter forceps length, especially in actual clinical practice in Japan where many patients have small bodies.

As for the difficulty of docking, it is essential for assistants to learn the technique through repeated training in attaching and detaching the forceps, but the docking technique itself is expected to be simplified by shortening the forceps length.

In summary, the lateral lymph node dissection performed using the Senhance Surgical System was both safe and precise, significantly contributing to reduced surgeon fatigue and enhanced patient recovery. With further refinement and accumulation of cases, the Senhance system has the potential to demonstrate new possibilities in robotic-assisted surgery and play a major role in the field of minimally invasive surgery. This report demonstrates the utility and potential of the Senhance Surgical System and contributes to the ongoing development of robotic surgery.

Surgical procedure for rectal cancer with Senhance in our department requires different directions of forceps docking for the central dissection and rectal transfer parts, as well as laparoscopically assisted rectal traction by an assistant. In the standard combined TME and lateral lymph node dissection (LLND) surgery, in addition to this, a left and right LLND must be performed. In this case, the right lateral dissection required a new 3-port incision, which means that at least 78 ports are needed to perform the same procedure. In addition, the expected significant increase in operating time makes the use of Senhance in combination with the standard TME and LLND procedure extremely unfeasible.

It is expected that the LUNA Surgical Robotic System, Senhance's next-generation digital surgical platform that also has a wider range of motion and articulated forceps currently under development, will make extended rectal cancer surgery possible in the future.

The number of cases in which only lateral lymph node dissection is performed in cases of lateral lymph node recurrence is small, and the degree of adhesion and the techniques used vary, we have not been able to compare the surgical results of this case with those of alternative techniques (laparoscopic surgery and da Vinci) in terms of operation time, blood loss, and postoperative recovery. This is one of the limitations of this report. Future comparisons based on the accumulation of cases will be necessary.

## CONCLUSIONS

We present the first lateral lymph node dissection performed using the Senhance Surgical System. We will accumulate more cases and study its usefulness in the future.

## DECLARATIONS

### Funding

None.

### Authors’ contributions

YM and YH wrote the manuscript.

YH supervised this report.

All authors have read and approved the manuscript.

### Availability of data and materials

Data sharing is not applicable to this article, since datasets were neither generated nor analyzed for the case series.

### Ethics approval and consent to participate

This work does not require ethical considerations or approval. Informed consent to participate in this study was obtained from the patient.

### Consent for publication

Written informed consent was obtained from the patient for the publication of this report.

### Competing interests

One of the authors, YH, received lecture fees as a proctor of Senhance system. The other authors have no competing interests.
